# Evaluation of Suitable Internal Control Genes for RT-qPCR in Yak Mammary Tissue during the Lactation Cycle

**DOI:** 10.1371/journal.pone.0147705

**Published:** 2016-01-25

**Authors:** MingFeng Jiang, Jung Nam Lee, Massimo Bionaz, Xiao Yu Deng, Yong Wang

**Affiliations:** 1 College of Life Science and Technology, Southwest University for Nationalities, Chengdu, China; 2 National Research Foundation of Korea, Seoul, Korea; 3 College of Agricultural Sciences, Oregon State University, Oregon, United States of America; 4 Sichuan Key Laboratory of Conservation and Utilization of Animal Genetic Resources in Tibetan Plateau, Southwest University for Nationalities, Chengdu, China; University of Tennessee Health Science Center, UNITED STATES

## Abstract

The yak is primarily found throughout the Tibetan high plateau and the surrounding mountainous area of south central Asia; among its others attributes, its milk is very important for the local population. A key concern in the field of yak research is the better understanding of which genes control the production and composition of milk. The most accurate and sensitive method for gene expression analysis is quantitative reverse transcription polymerase chain reaction (RT-qPCR). It is essential for reliable RT-qPCR to be able to the normalize the data using internal control genes (ICGs). However, it is critical to assess the reliability of the normalization by testing multiple ICGs. Our objective was to uncover a reliable normalization for RT-qPCR data obtained from yak mammary tissue during the lactation cycle. We assessed the reliability of 10 ICGs (*ACTB*, *EIF6*, *GAPDH*, *LRP10*, *MRPL39*, *MRPS15*, *MTG1*, *RPS8*, *RPS23*, and *UXT*) using geNorm. The analysis revealed that all of the tested ICGs can be considered to be reliable, but the use of the 6 most stable ICGs should be applied to yield a reliable normalization factor (NF). We compared the results of 3 target genes (*CSN1S1*, *ESR1*, and *MYC*) normalized using 6, 3, or 1 of the best ICGs. We did not observe overall differences between the 3 normalization strategies with the exception of 1 time point in *MYC*. The use of only a single ICG is not recommended; thus, we concluded that the calculation of the NF using the 3 best ICGs, *MRPS15*, *RPS23*, and *UXT*, is a reliable normalization strategy for RT-qPCR data obtained from yak mammary tissue during pregnancy and lactation. A dilution effect of the ICGs due to a large increase in the mRNA of abundantly expressed genes in bovine and porcine mammary tissue during the lactation cycle was previously observed. To test for the presence of a dilution effect in our study, we evaluated the pattern of non-normalized RT-qPCR data of ICGs from pregnancy to lactation and compared them with the total RNA concentration, milk yield, and non-normalized RT-qPCR data of 3 target genes. With a few exceptions, the non- normalized RT-qPCR data for the tested ICGs was significantly increased by lactation and had a positive correlation with total RNA and the non-normalized RT-qPCR data of *CSN1S1*. These data clearly indicated the presence of a “concentration effect” of single mRNA that remains unexplained but needs to be accounted for during the normalization of RT-qPCR data. Based on our findings, we recommend that the NF of the *MRPS15*, *RPS23*, and *UXT* genes should be used in the normalization of RT-qPCR data obtained from mammary tissue of lactating yaks during pregnancy and lactation.

## Introduction

The yak (*Bos grunniens*) is found throughout the Tibetan plateau of western China at high altitudes from 2000–5000 m where a severe ecological environment exist. The yak can use the pasture resources of this area, where few other domestic animals could survive and provides milk, meat, hair, and cheese to the regional people. The yak also transports goods across mountain passes for local farmers and traders and is also used in climbing and trekking expeditions. For these reasons, the yak is one of the most important domestic animals in Tibetan life [1] and the yak industry has grown rapidly in recent years [2].

Yak milk is commercially important to 6.5 million Tibetan people who drink it daily. For this reason, there is a need to better understand the molecular aspects of milk synthesis in yaks in order to improve milk production. The use of the data generated from high-producing dairy cows presents limitations in this regard. Despite being considered bovine, yaks differ substantially from dairy cows. Compared to high producing Holstein dairy cows, yaks have a very limited milk yield (in general less than 3 kg/day compared to 30 kg/day in Holsteins), and the composition of the yak milk is substantially different from that of Holstein cows, as it has a higher protein and fat content [3]. A key concern in the field of yak research is to better understand which genes control the production and composition of milk, how these genes are regulated, and how they might be manipulated to enhance milk yield, manufacturing properties, and health characteristics.

Among several methods used to analyze the expression of genes, RT-qPCR is the most accurate [4–5]. Essential for RT-qPCR accuracy is the correction for errors arising from sample preparation and processing, including the quantity of initial RNA, cleaning processes, and cDNA synthesis [6–8]. Such a correction is denoted as “normalization” [9–10]. Among several proposed methods to normalize RT-qPCR data, the gold standard is the use of ICGs, also called reference genes. The expression of proper ICGs must be unaffected by the conditions studied (i.e., the copy number of the transcript/cell has to remain constant). According to the minimum information for the publication of RT-qPCR data (MIQE) [11], the selection of ICGs is an essential step in RT-qPCR for each experiment. ICGs must be validated using specific algorithms and multiple ICGs must be considered.

The problem of selecting proper ICGs becomes even more pressing when time course experiments in tissues experiencing a significant biological change are considered. This was obvious in a study conducted in bovine mammary tissue from pregnancy to the end of the subsequent lactation [12]. In that case, a decrease in the raw (i.e., non-normalized) RT-qPCR data of several housekeeping genes was observed from pregnancy to lactation. The negative correlation of the increased total RNA/mg tissue and the raw RT-qPCR data for lactalbumin, one of the most abundant mammary transcripts that shows an extremely large increase in expression during lactation, demonstrated a dilution of the raw RT-qPCR data of stably expressed genes (i.e., “dilution effect”). The increased amount of total RNA has been suggested to be a consequence of the large increase in the expression of a relatively low number of highly abundant genes [12]. This phenomenon was also confirmed in the mammary glands of lactating pigs [13] and could also be expected as a result of the longitudinal gene expression experiment in the mammary tissue of yaks. Therefore, suitable ICGs should be selected to correct for a potential dilution effect.

In most studies performed in mammary tissue or mammary epithelial cells of yaks, the normalization of RT-qPCR data is typically performed using previously proposed ICGs for dairy cows or other species, without proper validation. Recently, the validation of ICGs to normalized RT-qPCR data from the somatic cells of yak milk was performed [14], but no ICGs have been validated for the normalization of RT-qPCR data generated from the mammary tissue of yaks. Thus, the selection of ICGs by biopsy of yak mammary tissue during whole lactation is indeed novel and this study allows researchers to determine the quality of data using RT-qPCR in the yak lactation research which is a relatively new field of research. Besides, there is still a large disregard for proper assessment of ICGs among scientists. This seems to be more the case in established fields of research, as partly indicated by the work of Bustin et al. [15] where a negative correlation was observed between compliance with MIQE guidelines [11] and impact factor of the journal. Thus, there is still the need for studies such as the present to clarify choice of loading controls during lactation per individual species in order to quantitate changes in gene expression.

For this reason, the objective of the present paper was to discover reliable ICGs for longitudinal gene expression studies of yak mammary tissue during the entire lactation cycle.

## Materials and Methods

This study was approved by the Southwest University for Nationalities Institutional Animal Care and Use Committee (permit number: 2011-3-2). The surgical procedure for yak mammary biopsy was carried out in strict accordance with the operations guide to ameliorate animal suffering. Five healthy female yaks in Hongyuan county of Sichuan Province in China were used. Mammary tissue samples (approximately 1 g) were collected by biopsy of the right or left rear quarters at -15, 1, 15, 30, 60, 120 and 240 days relative to parturition (d) as previously described [12]. All samples were immediately frozen and stored in liquid nitrogen. The milk yield for each yak was recorded during the entire lactation cycle (15, 30, 60, 120, and 240 d).

Part of the yak mammary tissue sample was weighed (50–100 mg) and immediately homogenized in 1 mL TRIzol (Invitrogen, Germany). RNA was extracted and the purity and concentration of RNA were determined by UV/Vis spectrophotometer (Eppendorf, Germany). The 260/280 ratio of RNA was ≥ 1.9. The integrity of RNA was assessed using 1% gel electrophoresis. All samples had a clear presence of the 2 expected bands at 18s and 28s without any evident of degraded products. The RNA was then diluted to 200 ng/μl using DNase and RNase-free water and 600 ng RNA were used for genomic DNA removal by PrimeScriptRT reagent Kit with gDNA Eraser (Takara Bio, Japan). The DNA-free RNA obtained was diluted with an equal amount of DNase and RNase-free water prior to cDNA synthesis. The cDNA was synthesized using PrimeScriptRT reagent Kit (Takara Bio, Japan) following the manufacturer’s instructions. The kit included a blend of oligo-dT and random hexamer primers for reverse transcription. The synthesized cDNA was diluted 1:3 with DNase and RNase-free water prior to RT-qPCR.

Ten genes were tested as potential ICGs: *ACTB* (β-actin), *GAPDH* (glyceraldehyde-3-phosphate dehydrogenase), *MTG1* (mitochondrial GTPase 1 homolog), *EIF6* (integrin-4 binding protein), *LRP10* (lipoprotein receptor-related protein 10), *MRPL39* (mitochondrial ribosomal protein L39), *MRPS15* (mitochondrial ribosomal protein S15), *RPS8* (ribosomal protein S8), *RPS23* (ribosomal protein S23), and *UXT* (ubiquitously expressed transcript isoform 2). The tested ICGs were selected based on previous published studies [12, 16–17] with the exception of *RPS8*. See Table 1 for a summary of the selected ICGs used in the present work and the use of those same ICGs in mammary or milk somatic cells in bovine, zebu, or yak in prior works. In addition, *ESR1* (estrogen receptor 1) and *MYC* (myelocytomatosis viral oncogene homolog) were selected as target genes following a previous publication [12], and *CSN1S1* (casein alpha s1) was selected based on its high abundance in mammary tissue. Casein alpha S1 comprises the major protein fraction of bovine milk (approximately 80%) [18].

**Table 1:** Summary of prior testing in bovine mammary tissue or milk somatic cells in several *Bos* species of selected internal control genes also tested in the present work.

ICG	*BOS INDICUS*	*BOS TAURUS*	*BOS GRUNNIENS*
	MILK SOMATIC CELLS	MAMMARY TISSUE	MILK SOMATIC CELLS
	*Varshney et al*, *2012*	*Bionaz and Loor*, *2007*	*Bai et al*, *2014*
***ACTB***	X*	X	X
***GAPDH***	X*	X	X
***MTG1***^#^	X	X	X
***EIF6***^$^		X	
***MRPL39***		X	X*
***MRPS15***	X	X*	X
***RPS23***		X	
***UXT***		X*	X*

*Used in the work to normalized target genes (i.e., best internal controls)

^#^Also reported as *GTP*

^$^Also reported as *ITGB4BP*

Primers for RT-qPCR were designed using Beacon Designer 7.6 software by fixing the amplicon size (bp) at 80–150 bp and the melting temp between 55 and 75°C. The sequences of selected genes were obtained from NCBI (http://www.ncbi.nlm.nih.gov/) and UCSC’s Cow (*Bos taurus*) Genome Browser Gateway (http://genome.ucsc.edu/). Prior to PCR analysis, each primer pair was tested to determine the optimal annealing temperature by gradient PCR. The product of each primer pair was verified by electrophoresis analysis on a 2% agarose gel to check for amplicon size and the absence of primer-dimers. The specificity of the amplicon was also verified by the presence of a single peak during the dissociation protocol (S1 Fig). The amplicon for each primer pair was also purified, sequenced by a 3730 DNA analyzer (ABI, USA), and the sequence was confirmed in BLAST against all possible transcript sequences in NCBI (S1 File). Information on PCR primer sets is summarized in Table 2.

**Table 2:** Characteristics of the primer pairs used and the efficiency of amplification.

Gene^1^	GenBank#	Primer sequence (5’to3’)^2^	AnnTemp^3^	bp	%E^4^
*ACTB*	AY141970	F: TCTTCGCCTTAATACTTGT	57.6	100	104
		R: AAGCCTTCATACATCTCAA			
*EIF6*	BU543794	F: GAGGGCTGGTACATCCCAAG	62.3	101	102
		R: CTCGCTGCCTCGGTTCAC			
*GAPDH*	BC102589	F: ACACTCACTCTTCTACCTTC	55.0	100	91
		R: TTGCTGTAGCCAAATTCATT			
*LRP10*	BC149232	F: CCAGAGGATGAGGACGATGT	62.2	139	98
		R: ATAGGGTTGCTGTCCCTGTG			
*MRPL39*	BC122667	F: AGGTTCTCTTTTGTTGGCATCC	59.0	101	98
		R: TTGGTCAGAGCCCCAGAAGT			
*MRPS15*	NM_001192201.1	F: GCAGCTTATGAGCAAGGTCGT	62.3	151	93
		R: GCTCATCAGCAGATAGCGCTT			
*MTG1*	NM_001025327	F: CTTGCTCGTCCTCAACAA	57.3	83	103
		R:TTATGCCTTCTCTTTCTAAGTGT			
*RPS8*	FG588970.1	F: CGAGTTCTATCTGAGGAA	51.8	85	100
		R: AAACGCCTTTATTAGATGA			
*RPS23*	NM_001034690.1	F: AATGATGGTTGCTTGAAT	56.0	169	104
		R: ATCTTGGTCTTTCCTTCT			
*UXT*	BC108205.1	F: TGTGGCCCTTGGATATGGTT	58.1	101	101
		R: GGTTGTCGCTGAGCTCTGTG			
*ESR1*	AY656813	F: GAGGAAGTGTAGTCATTG	57.6	116	102
		R: AGTTGGATTATCAGTTAGC			
*MYC*	BC113343	F: TACATCCTGTCGGTCCAA	57.6	103	100
		R: AACTGTTCTCGCCTCTTC			
*CSN1S1*	NM_00181029.2	F: GGGGAGTGAATCAGGAAC	54.2	154	99
		R: CCAATGGGATTAGGGATG			

^1^Gene symbol

^2^ F = Forward primer, R = Reverse primer

^3^Annealing temperature in RT-qPCR

^4^% E = percent PCR efficiency, where % E = (10^−1/Slope^-1)× 100%

PCR was carried out in triplicate for each sample in a CFX96 Real-time system (BIO-RAD, USA). A six-point standard curve was generated for each gene using a 10-fold dilution of cDNA to determine the efficiency of amplification for each primer pair. The PCR was performed in a 10 μl final volume containing 2 μl cDNA, 5 μl SsoFast EvaGreen supermix (BIO-RAD, USA), 0.5 μl each of 10 μM forward and reverse primers, and 2 μl DNase and RNase-free water. The instrument was set at 95°C for 10 min (enzyme activation), 40 cycles at 95°C for 15 s (denaturation), followed by the optimal annealing temperature of each primer (55~63°C, Table 2) for 1 min (annealing and extension), 95°C for 15 s, plus 65°C to 95°C for 15 s (melting curve). A negative control without the cDNA template was included in each assay.

The Relative Quantity (RQ) of each gene was calculated using a modified Pfaffl equation [19]:

$${\text{RQ}}_{\text{sample}}\left({\Delta \text{C}}_{\text{t}}\right)=E^{(\text{Ct}\left(\text{MIN}\right)-\text{Ct(Sample))}},$$

where C_t_ (MIN) = C_t_ for the analyzed sample with the lowest C_t_ (i.e., higher mRNA abundance) among all samples across all time points, C_t_ (Sample) = C_t_ for the sample, and *E* (Efficiency) = (10^−1/Slope^). The RQ of target genes was normalized by dividing the RQ data by the normalization factor (NF). The NF was calculated using the geometric mean of the RQ data of selected ICGs [19].

The geNorm algorithm proposed by Vandesompele et al. [9] (version 3.5, http://medgen.ugent.be) was used to evaluate the stability of the 10 ICGs and to determine the optimum number of ICGs to calculate a reliable NF. The geNorm program assesses the stability of candidate ICGs by performing a pair-wise comparison of the non-normalized RT-qPCR data. The geNorm algorithm provides the expression stability value (M), which indicates the stability of one ICG with respect to all the others, and the pair-wise variation value (V), which indicates the reliability of the NF obtained by using the best or most stable ICGs. According to Vandesompele et al. [9], an M value below 1.5 and a V value less than 0.15 are considered acceptable and indicate stable ICGs and reliable NF, respectively.

The fundamental rationale of the geNorm algorithm is that the larger the stability of the raw RT-qPCR data between two non-co-regulated ICGs across the samples, the higher the likelihood that these are stably expressed and thus are highly reliable ICGs. It is therefore critical that prior to geNorm analysis, that the absence of co-regulation among potential ICGs is verified, which otherwise biases geNorm analysis. The co-regulation analysis among candidate ICGs was performed using Ingenuity Pathway Analysis (IPA, Ingenuity Systems, USA, www.ingenuity.com). The analysis revealed that *ACTB*, *GAPDH*, *RPS23*, and *UXT* were all potentially co-regulated by *MYC*. The other ICGs (*EIF6*, *LRP10*, *MRPL39*, *MRPS15*, *MTG1*, and *RPS8*) have no known co-regulation (S2 Fig), The database used by IPA includes exclusively information generated in monogastrics (mainly mice, rats, and humans) and does not include ruminants data; therefore, the lack of co-regulation revealed by IPA has to be considered putative. Therefore, the geNorm analysis can potentially be biased by the co-regulation of 4 tested ICGs but most of the ICGs tested did not present co-regulation.

## Results and Discussion

### *MRSP15* is the best single ICG but 6 ICGs provide the best normalization strategy

**Fig 1:**
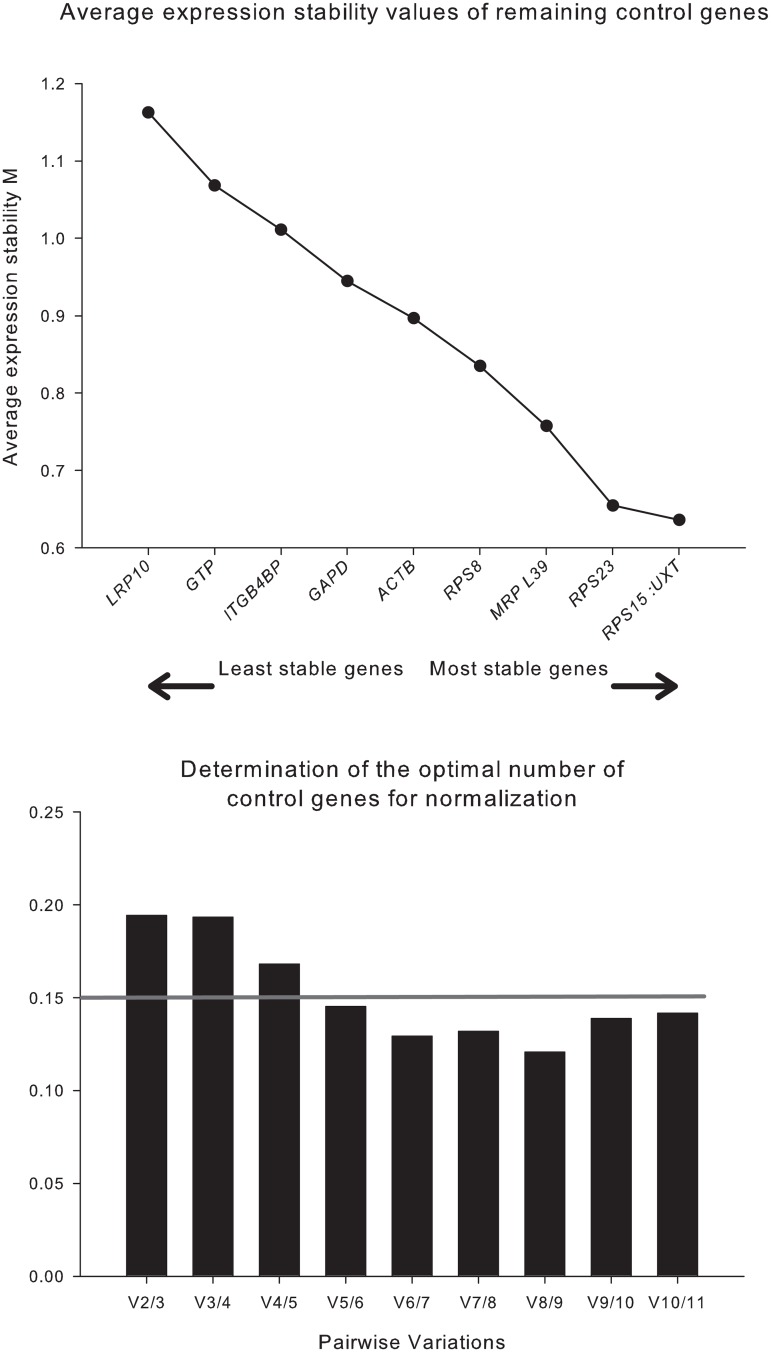
Average expression stability values (M value) and optimal number (V value) of ICGs for a reliable normalization factor for the 10 ICGs tested. The M values of ICGs in the mammary tissue of yaks under different time points were calculated using geNorm software. The proposed threshold is < 1.5 [9]. The V-value from geNorm indicates the reliability of the normalization factor; the lower the V-value, the higher the stability. The y-axis indicates pairwise variation V (V_n_/V_n+1_) between the calculation of the normalization factor (NF) using the best internal control genes (NF_n_) and the addition of the next “best” ICG (NF_n+1_). The proposed threshold for a reliable NF is a V value ≤ 0.15.

The rank based on the M value of all ICGs in yak mammary tissue is reported in Fig 1. The results indicate that the M value of *MRPS15* and *UXT* was the lowest (M = 0.64), i.e., the most stable among the ICGs tested; the M value of *LRP10* was the highest (M = 1.16), i.e., the least stable ICG, although it was still acceptable due to M-value < 1.5. To determine the optimal number of ICGs, the V value was calculated using geNorm software. According to the threshold proposed by Vandesompele et al. [9] (i.e., V ≤ 0.15), the results indicated that the use of 6 ICGs (V_5/6_ = 0.145) provided the most reliable NF for RT-qPCR data in the mammary tissue of lactating yaks when measured during the course of lactation (Fig 1). The potential co-regulation suggested by IPA (S2 Fig) had an absent or very minimal effect on the geNorm analysis because only 2 out of 4 potentially co-regulated ICGs were among the most stable (Fig 1) and the lowest pair-wise variation was observed between two genes that were not co-regulated (i.e., *UXT* and *MRPS15*).

### No differences are observed between the RT-qPCR results of target genes when using 3 or 6 ICGs

**Fig 2:**
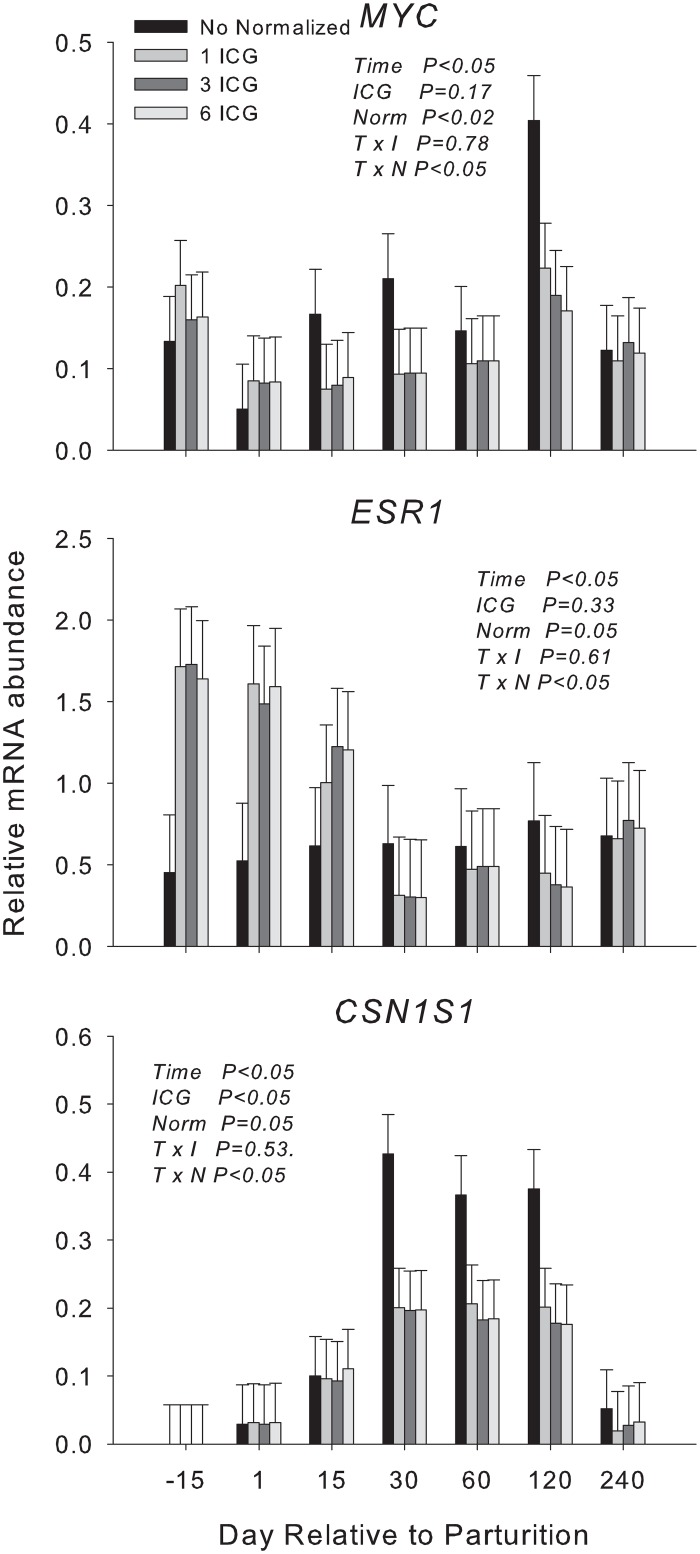
Comparison of the 3 normalization strategies (i.e., using 1, 3, or 6 ICGs) selected according to the M-value plus non-normalization. The least square means of the normalized RQ of 3 target genes: v-myc avian myelocytomatosis (*MYC*), estrogen receptor 1 (*ESR1*), and casein alpha s1 (*CSN1S1*) are shown. Error bars indicate standard error. The legend inside the figures denotes the significance of the effect analyzed with Time = the effect of the stage of lactation; ICG = the effect of the use of 0, 1, 3, or 6 ICG to normalize the raw RT-qPCR data; Norm = the effect of normalization (i.e., yes or no); and T × I = Time × ICG; T × N = Time × Norm.

Vandesompele et al. [9] indicated that the proposed V value of 0.15 was somewhat arbitrary. For this reason, instead of using the 6 best ICGs to normalized the RT-qPCR data, we tested the effect of normalizing RT-qPCR data using an NF calculation of the 3 best ICGs, which provided a V value of 0.193 (Fig 1). In addition to the above comparison we also assessed the effect of normalization vs. non-normalization and the effect of normalization employing the NF calculated using the best ICGs. To this end, we compared the RT-qPCR data of *ESR1*, *MYC*, and *CSN1S1* that were either non-normalized or normalized using the NF calculated using the best ICG (i.e., *MRPS15*), the 3 best ICGs (i.e., *MRPS15*, *UXT*, and *RPS23*), and the 6 best ICGs (i.e., *MRPS15*, *UXT*, *RPS23*, *MRPL39*, *RPS8*, and *ACTB*). The results are reported in Fig 2. The data were analyzed using the GLIMMIX procedure of SAS (v 9.4, SAS Institute Inc.) with two different analyses. One analysis included in the model the Time points and the Number of ICGs (none, 1, 3, or 6) and the interaction Time points × Number of ICGs as fixed effects. The other analysis included Normalization (Yes, No) and Time points × Normalization as fixed effects. In both analyses, Yak was considered a random effect. LSmeans were separated by Tukey’s test. We observed a significant effect (P < 0.05) of the normalization in all target genes but we did not observe any difference between the use of 1, 3, or 6 ICGs to calculate the NF (Fig 2). In addition, the NFs calculated using 3 and 6 ICGs was strongly correlated (r = 0.94, P < 0.01) (S3 Fig). The correlation between the calculated NFs using 3 or 6 ICGs with the NF calculated using the best ICG (i.e., *MRPS15*) was still significant, but the Pearson coefficient was lower compared to the correlation between the NF calculated using the 3 best ICGs and the NF calculated using the 6 best ICGs (r = 0.80 for the comparison of 1 ICG and 3 ICGs and r = 0.74 for the comparison of 1 ICG and 6 ICGs: S3 Fig). These results therefore indicated that the NF calculated using the 3 best ICGs (i.e., *MRPS15*, *RPS23*, and *UXT*) could provide as reliable an RT-qPCR normalization as with the NF calculated using the 6 best ICGs in longitudinal expression studies of mammary genes in lactating yaks. The normalized RT-qPCR data of target genes (Fig 2) showed that the expression of all target genes after normalization was significantly affected by time with the expression of *ESR1* and *MYC* being down-regulated and the expression of *CSN1S1* being up-regulated from 15 to 120 compared to -15 d. These results are somewhat similar to those of previous reports [3,12].

### No dilution effect was observed among stably expressed genes

Analyses of ICGs in the mammary tissue of bovine and porcine from pregnancy to the end of lactation strongly indicated the presence of an artificial “dilution effect” of stably expressed genes [12–13]. To test the presence of a dilution effect in our data, we compared the NF calculated using the 10 ICGs (corresponding to the overall temporal pattern of non-normalized RT-qPCR data of the 10 ICGs) with the pattern of total μg RNA/mg tissue and milk yield. Data were checked for normal distribution using the Proc Univariate of SAS and parameters with a significant (i.e., P < 0.05) Shapiro Wilk test were log_2_ transformed before statistical analysis. The statistical analysis of non-normalized expression data, RNA concentration, and milk yield was performed using the GLIMMIX procedure in SAS (v 9.4, SAS Institute Inc.) with the main effect being time relative to parturition and with Yak as a random effect. The LSmeans data between time points was separated by Tukey’s test. Significance was declared at P < 0.05. In addition, we also have performed a Pearson correlation between the non-normalized RQ of each ICG, RNA concentration, milk yield, and non-normalized RQ of target genes using the CORR procedure in SAS (S2 File).

**Fig 3:**
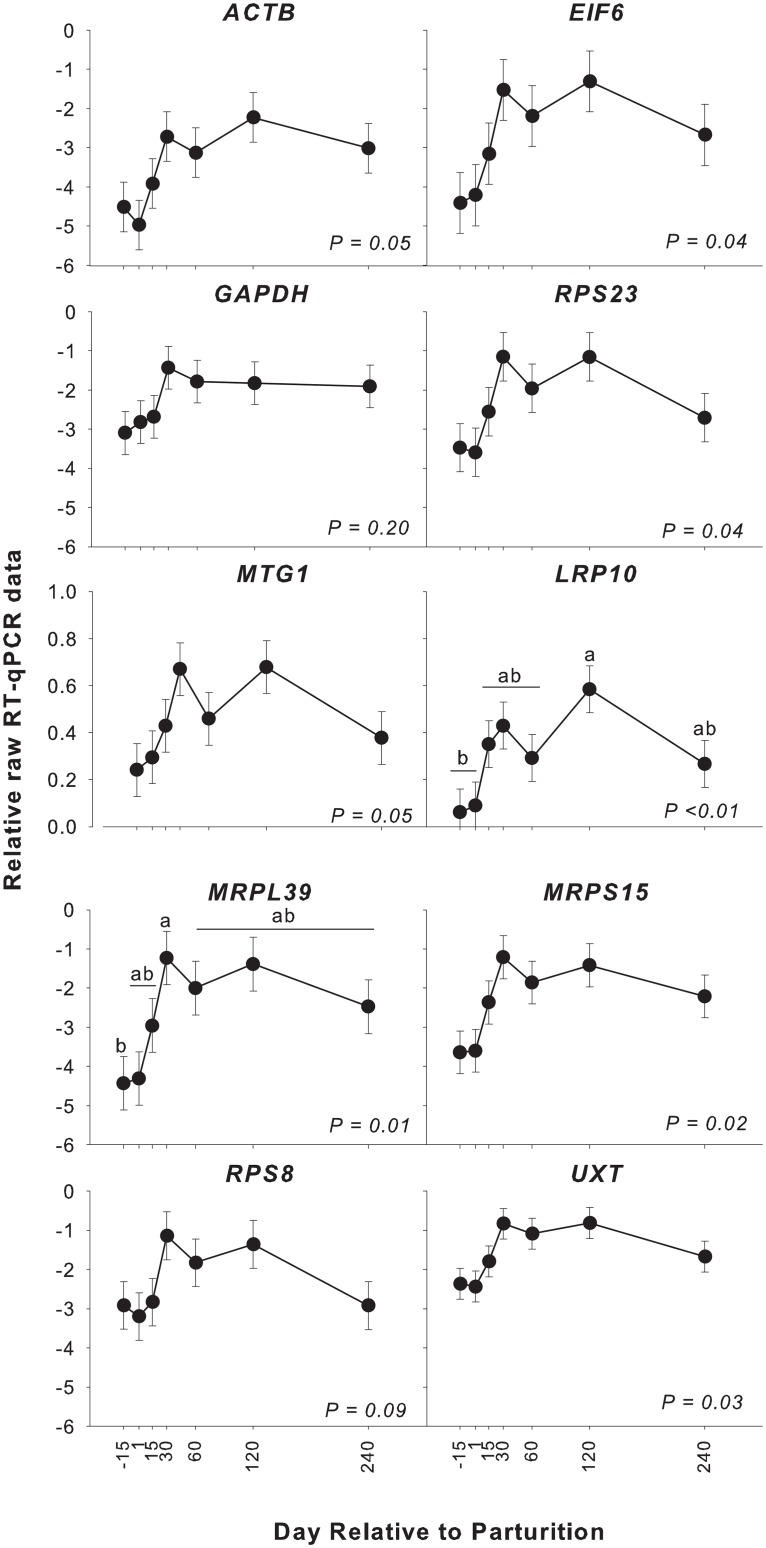
The expression pattern of the non-normalized RT-qPCR data for the 10 ICGs tested during the lactation cycle in yak mammary tissue. P-values were obtained using GLIMMIX analysis in the SAS program. The raw RQ data of all ICGs, with the exception of *MTG1* and *LRP10*, were log_2_ transformed prior to statistical analysis. LSmeans ± SE are shown. Different letters denote a significant difference between time points after post-hoc correction using Tukey’s method.

**Fig 4:**
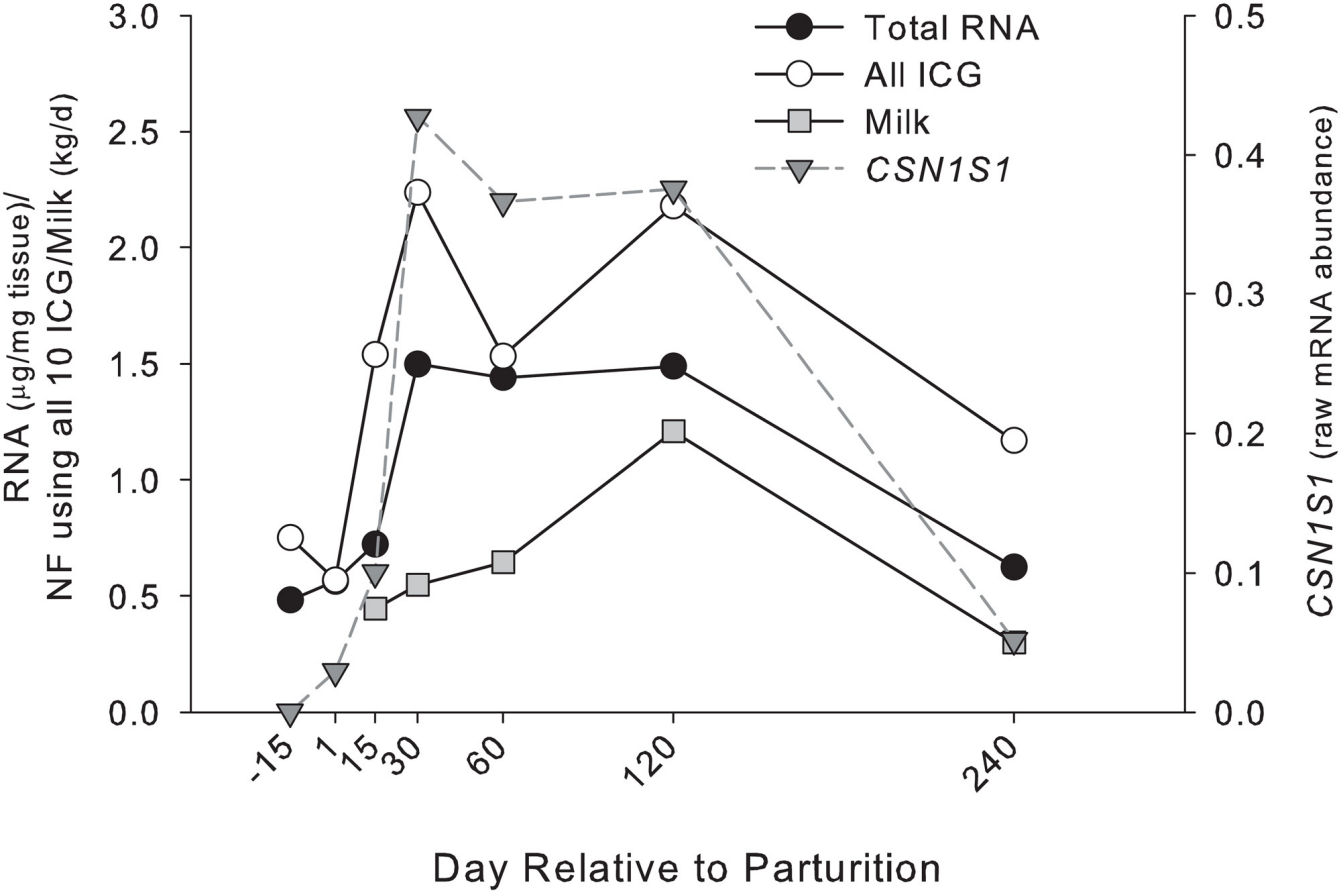
The normalization factor pattern during the lactation cycle in yak mammary tissue using all 10 ICGs, including total μg RNA/mg tissue, milk yield, and non-normalized RT-qPCR data for *CSN1S1*. All parameters were affected by time (P < 0.05).

The temporal pattern of non-normalized RQ data was significantly affected by time in all tested ICGs with the exception of *GAPDH* and *RPS8* (Fig 3). In addition, we observed a significant positive correlation (P < 0.05) between the RQ data of *MTG1*, *LRP10*, and *UXT* with RNA concentration and, with the exception of *GAPDH* and *ACTB*, we also observed a strong positive correlation between the tested ICGs and the non-normalized RQ of target genes, including *CSN1S1* (S2 File). This positive correlation can also be seen by the direct pattern of RNA concentration, milk yield, and NF calculated using all ICGs (Fig 4).

The above data disagree substantially with previous observations in bovine and porcine of a dilution effect of stably expressed genes [12–13]. Our results strongly indicated that the dilution effect was not present in the tested ICGs during the lactation cycle in yak mammary tissue. Rather, the data indicated a “concentration effect”. The “concentration effect” is the opposite of a “dilution effect”, i.e., the raw or non-normalized RQ data of stably expressed genes appear to be up-regulated through time due to an artificial increase in the relative concentration of mRNA in the total RNA used for RT-qPCR analysis, but in reality, the copy number/cell is constant. We can postulate that this “concentration effect”, if actually present, was the consequence of a decrease in the expression of relatively few very abundantly expressed genes (e.g., ribosomal RNA); however, the observed increase in RNA concentration does not seem to support such a conclusion. Another reason could be an overall large increase in the expression of mRNA relative to rRNA and tRNA. However, this is speculation and the presence of a “concentration effect” remains unexplained. It is important to note that a proper normalization should account for such a concentration effect. Interestingly, the lack of a dilution effect in the present experiment was similar to observations of yak milk somatic cells by Bai et al.[14]. However, in the work of Bai et al, milk somatic cells were used, which lacked a dry period sample. In the work of Bionaz and Loor [12], the largest difference in the pattern of raw (i.e., non-normalized) data of the ICGs was observed from -15 to 1 d. Thus, the lack of a dilution effect observed in Bai et al. can be partly explained by the lack of a dry period sample.

## Conclusions

The results from our study indicated that the use of 6 ICGs would provide the most reliable normalization among the 10 tested ICGs; however, a more detailed analysis suggested that using the 3 best ICGs (i.e., *MRPS15*, *RPS23*, and *UXT*) provided a normalization with the same reliability as the use of the 6 best ICGs. The most stable ICGs of yak mammary tissue uncovered in the present study were somewhat similar to the ICGs deemed reliable for bovine mammary tissue (i.e., *UXT*, *RPS9* and *RPS15*) [9], but were to some extent different than the ones uncovered to be the most reliable ICGs in the milk somatic cells of yaks (*RPS9*, *PPP1R11*, *UXT* and *MRPL39*) [14] and in the milk somatic cells of bovines (*PPP1R11*, *ACTB*, *UBC* and *GAPDH*) [20]. This highlights the unreliability of using previously proposed combinations of ICGs for the normalization of RT-qPCR data, even if the data were produced at the same time period of the experimental design in a different, but related, species (e.g., time course in dairy cows) or in a different type of sample in the same species (e.g., the milk somatic cells of yaks). Thus, it is essential to validate the reliability of the normalization strategy in each experiment using multiple ICGs.

Overall, the present study uncovered that the calculation of the NF using *MRPS15*, *RPS23*, and *UXT* is a reliable strategy for the normalization of RT-qPCR data in yak mammary tissue during the lactation cycle. These 3 ICGs can initially be used to test the reliability of the NF using geNorm or other algorithms in studies related to mammary tissue in lactating yaks.

## Supporting Information

S1 Fig Melting curves of the 10 ICGs used for RT-qPCR to validate the presence of a single peak for each primer pair. (PDF)

S2 Fig Co-regulation analysis of the 10 tested reference genes (purple shade) using Ingenuity Pathway Analysis (Ingenuity system, Germany, www.ingenuity.com). The potential up-stream regulator(s) are reported (no background) with the potential co-regulators highlighted by a light blue shade. Arrows denote an indirect (dashed) or direct effect. The cellular location of the proteins coded by the genes is also shown.
(JPG)

S3 Fig Correlation between the geometric mean using 1, 3, or 6 ICGs. The scatter plot for the geometric mean of each ICG as the normalization value is shown; r indicates the correlation coefficient, and P is the significance of the correlation.
(PDF)

S1 File Sequencing results for the validation of the 10 ICGs PCR products. (XLS)

S2 File Pearson correlation analysis using each 10 ICGs, total μg RNA/mg tissue, milk yield, and non-normalized RT-qPCR data for *CSN1S1*. (XLS)

## References

[1] Miller DJ, Harris RB, Cai CQ. Wild yak and their conservation in the Tibetan Plateau. In Proceedings of the First International Congress on Yak. 1994 Jun; 27–35

[2] Zhong JC, Zi XD, Han JL, Chen XH. Yak production in central Asian highlands. Proceedings of the fourth international congress on yak. 2004; 32–36

[3] Wiener G, Han JL, Long RJ. The Yak Second Edition. RAP publication 2006;119–120

[4] Hembruff SL, Villeneuve DJ, Parissenti AA. The optimization of quantitative reverse transcription PCR for verification of cDNA microarray data. Anal. Biochem. 2005;345: 237–249 16139235 10.1016/j.ab.2005.07.014

[5] Valasek MA, Repa JJ. The power of real-time PCR. Adv. Physiol. Educ. 2005; 29:151–159 16109794 10.1152/advan.00019.2005

[6] Andersen CL, Jensen JL, Orntoft TF. Normalization of real-time quantitative reverse transcription-PCR data: a model-based variance estimation approach to identify genes suited for normalization, applied to bladder and colon cancer data sets. Cancer Res. 2004; 64:5245–5250 15289330 10.1158/0008-5472.CAN-04-0496

[7] Bustin SA, Nolan T. Pitfalls of quantitative real-time reverse transcription polymerase chain reaction. J. Biomol. Tech. 2004; 15:155–166 15331581 PMC2291693

[8] Huggett J, Dheda K, Bustin S, Zumla A. Real-time RT-PCR normalization; strategies and considerations. Genes Immun. 2005; 6(4):279–284 15815687 10.1038/sj.gene.6364190

[9] Vandesompele J, De Preter K, Pattyn F, Poppe B, Van Roy N, De Paepe A, et al Accurate normalization of real-time quantitative RT-PCR data by geometric averaging of multiple internal control genes. Genome Biol. 2002; 3: RESEARCH0034 12184808 10.1186/gb-2002-3-7-research0034 PMC126239

[10] Dheda K, Huggett JF, Bustin SA, Johnson MA, Rook G, Zumla A. Validation of housekeeping genes for normalizing RNA expression in real-time PCR. Biotechniques 2004; 37: 112–119 15283208 10.2144/04371RR03

[11] Bustin SA, Benes V, Garson JA, Hellemans J, Huggett J, Kubista M, et al The MIQE guidelines: minimum information for publication of quantitative real-time PCR experiments. Clin. Chem. 2009; 55(4):611–622 10.1373/clinchem.2008.112797 19246619

[12] Bionaz M, Loor JJ. Identification of reference genes for quantitative real-time PCR in the bovine mammary gland during the lactation cycle. Physiol. Genomics. 2007; 29(3):312–319 17284669 10.1152/physiolgenomics.00223.2006

[13] Tramontana S, Bionaz M, Sharma A, Graugnard DE, Cutler EA, Ajmone-Marsan P, et al Internal controls for quantitative polymerase chain reaction of swine mammary glands during pregnancy and lactation. J. Dairy Sci. 2008; 91(8):3057–3066 10.3168/jds.2008-1164 18650282

[14] Bai WL, Yin RH, Zhao SJ, Jiang WQ. Selection of suitable reference genes for studying gene expression in milk somatic cell of yak (Bos grunniens) during the lactation cycle. J. Dairy Sci. 2014; 97:902–910 10.3168/jds.2012-6437 24342693

[15] Bustin SA, Benes V, Garson J, Hellemans J, Huggett J, Kubista M, et al The need for transparency and good practices in the qPCR literature. Nat. Methods. 2013; 10(11): 1063–1067 10.1038/nmeth.2697 24173381

[16] Wathes DC, Cheng Z, Fenwick MA, Fitzpatrick R, Patton J. Influence of energy balance on the somatotropic axis and matrix metalloproteinase expression in the endometrium of the postpartum dairy cow. Reproduction. 2011; 141(2):269–281 10.1530/REP-10-0177 21123519 PMC3021913

[17] Saremi B, Sauerwein H, Dänicke S, Mielenz M. Identification of reference genes for gene expression studies in different bovine tissues focusing on different fat depots. J. Dairy Sci. 2012; 95(6):3131–3138 10.3168/jds.2011-4803 22612949

[18] Chaiyabutr N. Milk production—Advanced genetic traits, cellular mechanism, animal management and health. InTech. 2012; 1: 4–5

[19] Pfaffl MW. A new mathematical model for relative quantification in real-time RT-PCR. Nucleic Acids Res. 2001; 29(9):e45 11328886 10.1093/nar/29.9.e45 PMC55695

[20] Varshney N, Mohanty AK, Kumar S, Kaushik JK, Dang AK, Mukesh M, et al Selection of suitable reference genes for quantitative gene expression studies in milk somatic cells of lactating cows (Bos indicus). J. Dairy Sci. 2012; 95(6)2935–2945 10.3168/jds.2011-4442 22612931

