# Supplementary figures and images for: Evaluation of Suitable Internal Control Genes for RT-qPCR in Yak Mammary Tissue during the Lactation Cycle
Source: PLoS One. 2016 Jan 25;11(1):e0147705. doi: 10.1371/journal.pone.0147705 (PMC4726593; doi:10.1371/journal.pone.0147705)

***ACTB***

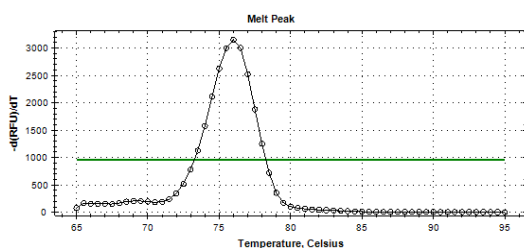

***EIF6***

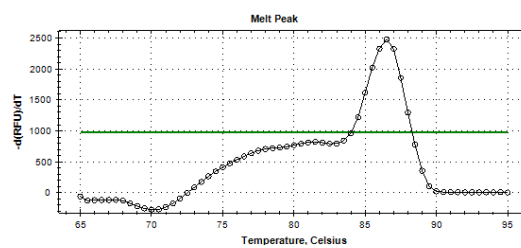

***GAPDH***

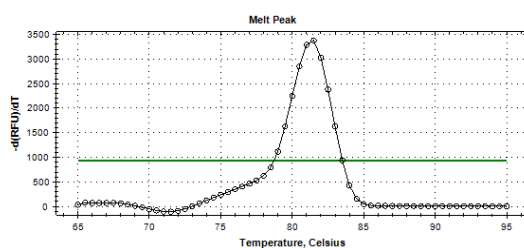

***LRP 10***

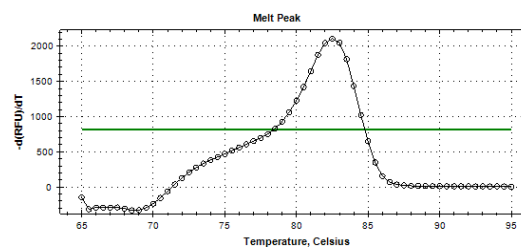

***MRPL39***

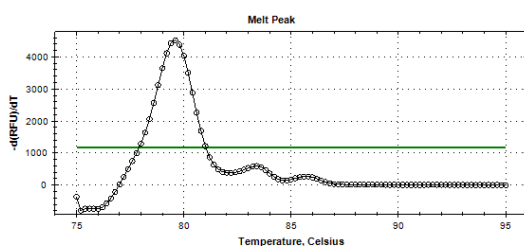

***MRPS15***

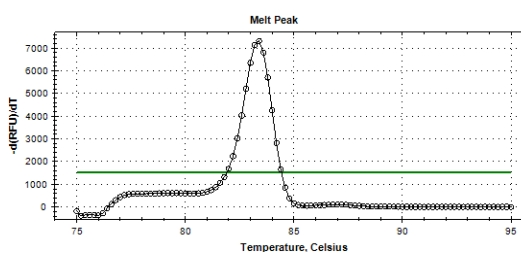

***MTG1***

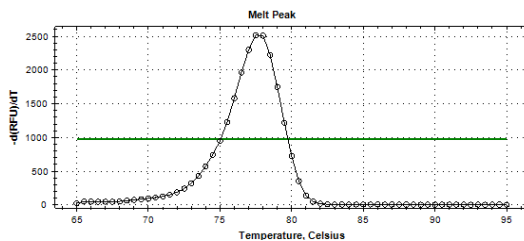

***RPS 8***

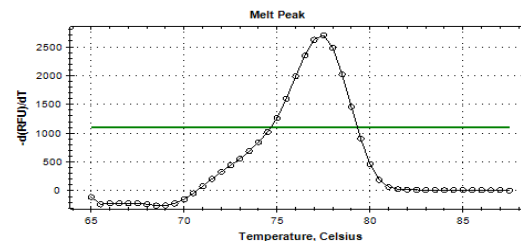

***RPS23***

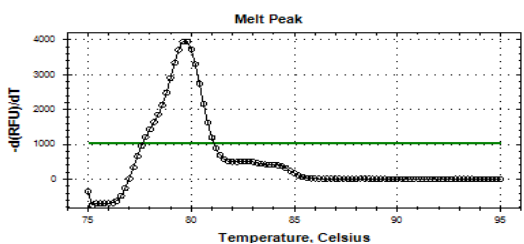

***UXT***

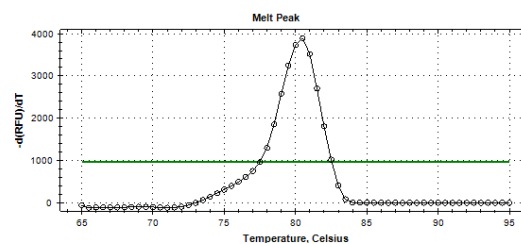

Supplement: S1 Fig — (PDF) [file pone.0147705.s001.pdf]

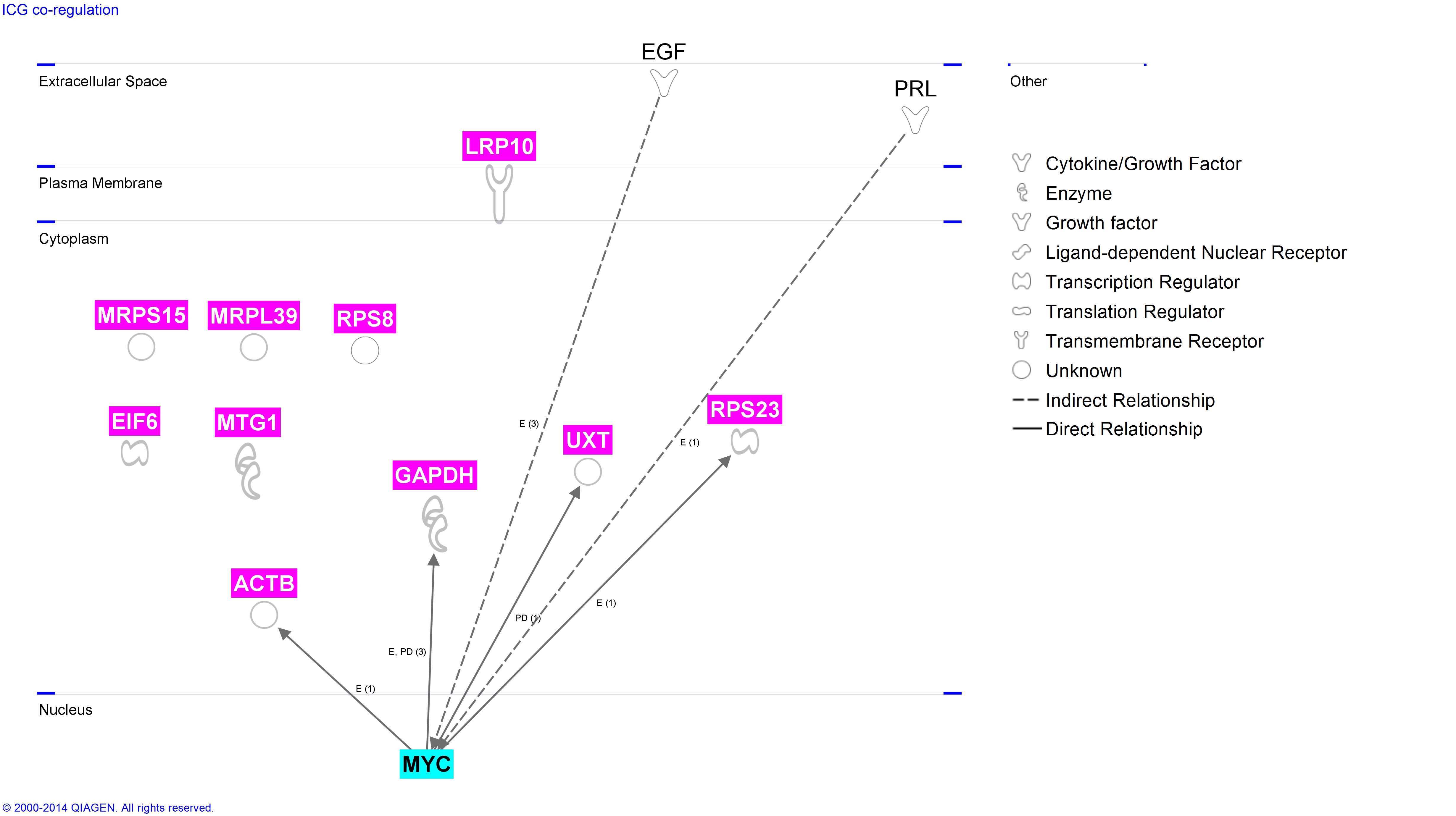

Supplement: S2 Fig — The potential up-stream regulator(s) are reported (no background) with the potential co-regulators highlighted by a light blue shade. Arrows denote an indirect (dashed) or direct effect. The cellular location of the proteins coded by the genes is also shown. (JPG) [file pone.0147705.s002.jpg]

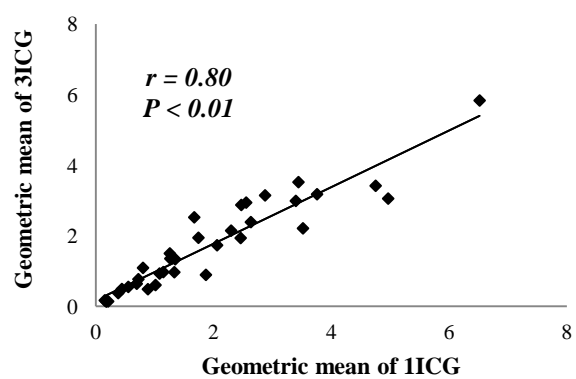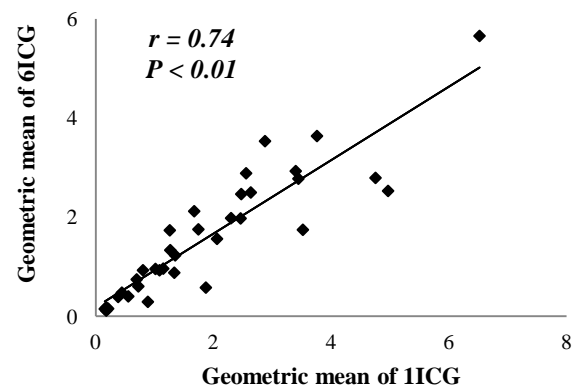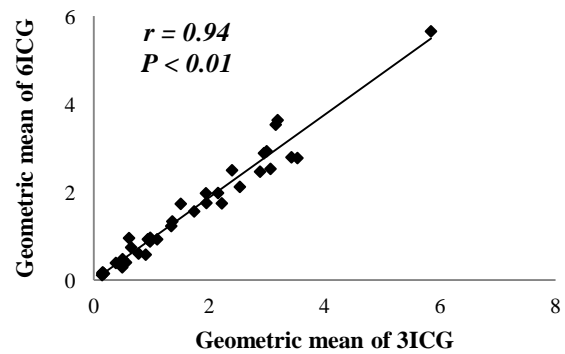

Supplement: S3 Fig — The scatter plot for the geometric mean of each ICG as the normalization value is shown; r indicates the correlation coefficient, and P is the significance of the correlation. (PDF) [file pone.0147705.s003.pdf]
